# Empathy in undergraduate medical students: A multivariate cross-sectional study in China

**DOI:** 10.1371/journal.pone.0336143

**Published:** 2025-11-05

**Authors:** Jiong Liu, Hong Duo, Yiwu Su, Cuijiao Tian

**Affiliations:** 1 Department of Radiation and Medical Oncology, Hubei Key Laboratory of Tumor Biological Behaviors, Hubei Province Cancer Clinical Study Center, Zhongnan Hospital, Wuhan University, Wuhan, Hubei, China; 2 Zhongnan Hospital of Wuhan University, Institute of Hepatobiliary Diseases of Wuhan University, Transplant Center of Wuhan University, Hubei Key Laboratory of Medical Technology on Transplantation, Wuhan, China; 3 Information Center of Zhongnan Hospital of Wuhan University, Wuhan, Hubei, China; 4 Teaching Affairs Office, Zhongnan Hospital of Wuhan University, Wuhan, Hubei, China; University of Tabuk, SAUDI ARABIA

## Abstract

**Context:**

Investigate the impact of various factors on medical students’ empathy scores, and provide constructive ideological and political education strategies to enhance their empathy abilities.

**Methods:**

This cross-sectional study involved 320 undergraduate medical students from Wuhan University, spanning three academic years. A web-based questionnaire assessed empathy, including the Jefferson Scale of Empathy-Student Version (JSE-S). Various factors, including grade, gender, medical major choice, urbanization level, clinical course participation, internship experience, number of romantic relationships, and experience of breakups, were examined for their influence on empathy scores.

**Results:**

There is a significant correlation between medical major selection, gender, and empathy scores. Furthermore, in binary logistic regression analysis, the influence of medical major selection on empathy scores surpasses that of the inherent variable, gender.

**Conclusions:**

This study indicates that medical major choice and gender are predictive factors of empathy ability among medical students. More large-sample studies or qualitative research should be conducted to explore the influencing factors of empathy ability in medical students, with the aim of further enhancing their empathy levels. Additionally, we found that students who initially did not choose a medical major and male students are groups with relatively lower empathy levels. Medical schools should focus on empathy education for these groups in future training programs, aiming to increase their interest in medicine and improve their empathy levels.

## 1 Introduction

The concept of empathy was initially discussed in 1873 by Robert Vischer, a German art historian and philosopher who utilized the term ‘Einfühlung’ to describe an observer’s emotional response evoked by artworks [1]. This concept has gradually extended into the field of medicine. Hojat et al. proposed the concept of empathy within the context of patient care, emphasizing that empathy is primarily a cognitive attribute (rather than an emotional or affective one). This attribute involves understanding the experiences, concerns, and perspectives of patients (rather than merely feeling them), the ability to convey this understanding, and the willingness to offer assistance [2]. The significance of empathy in the healthcare domain has gained increasing recognition in recent years. Empathy plays a vital role in fostering a satisfactory patient-physician relationship [3]. Studies have shown that empathy is linked to both academic performance and clinical competence among medical students [3], as well as to positive patient outcomes [4]. Furthermore, higher levels of empathy have been associated with increased self-esteem [5] and reduced burnout and distress [6,7].

Given the critical role of empathy in the medical profession, numerous countries have undertaken investigations into the empathy competencies of medical students [8–11]. A substantial body of literature has identified various factors associated with empathy levels among medical students, including gender, parental relationships, ethnicity, specialty preference, parental income, and academic year, among others [12–15]. Research on the relationship between gender and empathy is relatively well-established, with most studies indicating that female students tend to exhibit higher levels of empathy [12,16], although a limited number of reports have found no significant gender differences [17,18]. Academic year has also been shown to correlate with empathy, with students in earlier years generally demonstrating higher empathy than those in more advanced stages of training [8,16,19], though differences may vary across regions [20]. Furthermore, interest in specific medical specialties has been associated with empathy, as students inclined toward people-oriented specialties often show higher empathy levels compared to those interested in technology-oriented fields [10,14]. Although empathy among Chinese medical students has been explored in a handful of studies over the past five years [13,21,22], the scope of variables examined remains limited. To date, no studies have been identified that investigate the relationship between Chinese students’ initial medical major choice, romantic relationship experiences, and empathy levels.

Thus, it is imperative to develop a systematic understanding of the overall state of empathy among undergraduate medical students in China. Alongside this, a thorough analysis of key influencing factors is essential to inform targeted strategies for enhancement. This study investigates the effects of various factors on the empathy scores of medical students, aiming to provide constructive guidance for enhancing their empathy abilities.

## 2 Methods

### 2.1 Research approach

This study employed a cross-sectional observational research method, with the JSE-S questionnaire distributed between September 1 and September 3, 2023. The participants were 320 voluntary undergraduate medical students from the Second Clinical College of Wuhan University, spanning three academic years: 2018, 2019, and 2020. The cohort included 84 third-year students (Grade 2020), 105 fourth-year students (Grade 2019), and 131 fifth-year students (Grade 2018). The questionnaire covered eight variables: gender, academic year, medical major choice, urbanization level of home address, completion of clinical intensive training courses, participation in clinical internships, number of romantic relationships, and experiences of breakups. Medical major choice was operationalized as the choice of specialization made following China’s college entrance examination, categorized dichotomously as either voluntary (indicating a preference for medical studies) or involuntary (influenced by external factors such as familial pressure, peer influence, or institutional adjustments). To ensure data integrity, each student account was restricted to a single submission, and all questions in the questionnaire were mandatory—none could be skipped or left blank. Additionally, responses that showed logical inconsistencies in three or more pairs of forward- and reverse-scored items, or that exhibited obvious patterned responses, were excluded. The survey was closed after a 72-hour participation period, resulting in a total of 320 valid questionnaires collected, with three discarded due to patterned answering.

Considering the need for subsequent regression analysis, the sample size was determined using the rule of thumb [23], which suggests a sample size of 5–20 times the number of variables. With eight variables in this study, 160 participants would meet the most stringent rule of thumb, while the 320 participants in this study are sufficient to ensure the reliability and validity of the statistical analysis.

### 2.2 Instrument

The Jefferson Scale of Empathy-Student version (JSE-S) is a latent variable scale consisting of three factors: “perspective taking,” “compassionate care,” and “standing in the patient’s shoes.” [24] Due to its extensive global usage and solid psychometric foundation, the JSE is widely recognized as the most extensively investigated tool in medical education research and is regarded as the primary instrument for assessing empathy in medical education. The JSE has been translated into 56 languages and utilized in over 85 countries [2,25–27]. In this study, we employed the Chinese version of the JSE-S to evaluate clinical empathy among medical undergraduates. We obtained permission to use the Jefferson Scale from the Jefferson Medical College. Comprising 20 questions, this self-administered instrument includes ten positive and ten negative items. Response is scored on a 7-point Likert-type scale. For positive items, responses are directly scored (strongly disagree = 1, strongly agree = 7). For negative items, responses are reversely scored (strongly disagree = 7, strongly agree = 1).

### 2.3 Ethical procedure

Our study protocol was approved by the Ethics Committee of Zhongnan Hospital, Wuhan University (Approval No. 2023030K). All participants are adults and have provided informed consent in the questionnaire. A senior administrator was appointed to coordinate the survey distribution and supervise its collection, ensuring adherence to the study protocol. Prior to initiating the survey, students from various academic levels were informed about the project and educated on the significance of their participation through campus announcements. Furthermore, prior to distributing the online questionnaire, researchers elucidated the study’s objectives and emphasized maintaining anonymity throughout all stages of research. No personal identifiable information was collected, and stringent confidentiality measures were implemented for all individual data.

### 2.4 Statistical analysis

Before conducting data statistical analysis, we performed confirmatory factor analysis (CFA) and calculated the Cronbach’s Alpha coefficients for the entire scale and its three-factor structure to evaluate the reliability and validity of the scale comprehensively.

The collected data was input into our pre-designed spreadsheet and analyzed using R version 4.3.1 and IBM SPSS Statistics 25. Continuous variables were presented as means accompanied by standard errors (SE). Following descriptive analysis, comparisons of total empathy scores were made based on gender, grade, medical major choice, urbanization level, clinical course, internship, number of romantic relationships, and experience of breakups. Student t-tests were employed for comparing empathy scores based on gender, medical major choice, clinical course, internship status, and experience of breakups. Additionally, analyses of variance (ANOVA) were performed for grade, urbanization level and number of romantic relationships. Finally, two prediction models were established to predict students’ empathy levels, with the median empathy score of 320 students used as the threshold. A p-value less than 0.05 was considered statistically significant.

## 3 Results

### 3.1 Reliability and validity analysis

The internal consistency of the JSE data was assessed in this reliability analysis by computing Cronbach’s α. We calculated the overall Cronbach’s α coefficient, as well as the Cronbach’s α coefficients for three sub-scales. The overall coefficient is 0.89; the coefficients for the three sub-scales are 0.88, 0.88, and 0.82, respectively. This suggests that the internal consistency of the three-factor structure of the JSE scale, as well as the internal consistency of the overall scale, is relatively high.

The CFA analysis reveals that the factor structure of the Chinese version of the JSE-S scale we translated is consistent with that of the original English version. The standardized factor loading coefficients for items 18 and 15 are 0.37 and 0.47, respectively, indicating a moderate level of association with the corresponding second and first factors. The standardized factor loading coefficients for the remaining 18 items are all above 0.5, demonstrating a high level of association with their respective factors of the JSE-S. This suggests that the scale possesses good convergent validity. Additionally, the chi-square to degrees of freedom ratio is less than 3, the AVE (Average Variance Extracted) ranges from 0.46 to 0.70, and the CR (Composite Reliability) ranges from 0.82 to 0.89. These three indicators further support the scale’s good convergent validity. Other indicators, such as CFI (Comparative Fit Index), TLI (Tucker-Lewis Index), SRMR (Standardized Root Mean Square Residual), and RMSEA (Root Mean Square Error of Approximation), also demonstrate the scale’s good convergent validity. The correlation matrix also demonstrates good discriminant validity of the scale structure. In conclusion, the above data suggest that the overall validity of the scale is good (S1 File).

### 3.2 Empathy scores stratified by various variables

As shown in Table 1, only gender (p = 0.044) and medical major choice (p = 0.002) demonstrated statistically significant differences, while the other six variables did not show significant differences. Additionally, the effect size for medical major choice was 0.43, approaching a medium effect, whereas the effect size for gender was 0.23, indicating a small effect. In subsequent analyses, we will focus specifically on medical major choice and gender for further investigation.

**Table 1:** Empathy scores among participants stratified by various factors (n = 320).

Factor	No. of participants	Empathy score		Effect size*	P-value
		Mean ± standard deviation	95% confidence interval		
**Gender**					
Male	135	107.26 ± 15.57	104.61-109.91	−0.23	0.044
Female	185	110.66 ± 13.79	108.66-112.66		
**Grade**					
2020	84	109.44 ± 14.51	106.29-112.59	0.002	0.68
2019	105	110.07 ± 15.13	107.14-112.99		
2018	131	108.41 ± 14.40	105.92-110.90		
**Medical Major Choice**					
own decision^a^	248	110.60 ± 14.55	108.78-112.42	−0.43	0.002
others^b^	72	104.47 ± 14.03	101.17-107.77		
**Urban level**					
Big city^c^	44	111.25 ± 13.12	107.26-115.24	0.004	0.50
Small city^d^	190	109.29 ± 14.47	107.22-111.36		
Rural areas^e^	86	108.05 ± 15.76	104.67-111.43		
**Clinical courses** ^**f**^					
Participated	245	109.40 ± 14.66	107.55-111.24	0.050	0.71
Not participated	75	108.67 ± 14.68	105.29-112.04		
**Internship**					
Participated	244	109.34 ± 14.65	107.48-111.18	0.032	0.81
Not participated	76	108.87 ± 14.70	105.51-112.23		
**Number of romantic relationships**					
0	134	107.98 ± 15.64	105.30 - 110.65	0.010	0.55
1	77	109.44 ± 13.83	106.30 - 112.58		
2	61	110.57 ± 12.78	107.30 - 113.85		
3	32	109.09 ± 16.91	102.99 - 115.19		
> 3	16	113.75 ± 11.56	107.59 - 119.90		
**Experience of breakups**					
No	165	108.29 ± 15.15	105.97 - 110.63	−0.13	0.24
Yes	155	110.21 ± 14.06	107.34 - 112.44		

Note: The table presents the empathy scores (mean ± standard deviation) along with 95% confidence intervals for different participant groups based on various variables. a) voluntary selection and inclination towards a medical major; b) involuntary selection or no inclination towards a medical major (choices influenced by uncontrollable factors, such as family and peer recommendations, major adjustments); c) Participants residing in second-tier cities and above; d) Participants residing below the second-tier city but above the town level; e) Participants residing at the town level and below; f) Clinical courses refer to intensive clinical courses conducted by our school during the fourth year of undergraduate education. * The effect size for an independent samples t-test is reported as Cohen’s d. The effect size for analysis of variance is represented by the partial eta squared (η2).

### 3.3 Empathy scores across gender stratified by other variables

Firstly, the research results revealed that a significant difference in empathy scores was only observed between male and female students in the 2018 cohort (p = 0.004). Secondly, female students who participated in clinical courses (p = 0.011) and internships (p = 0.002) scored significantly higher in empathy than their male counterparts. Additionally, female students who had not experienced a breakup also had significantly higher empathy scores than males (p = 0.030), while no significant difference in empathy scores was found between males and females who had experienced a breakup. For other factors, no significant gender differences were observed (Table 2).

**Table 2:** Empathy scores across gender stratified by various factors (n = 320).

Factor	Gender	No. of participants	Empathy score		Effect size	P-value
			Mean ± standard deviation	95% confidence interval		
**Grade**						
** **2020	**male**	33	109.81 ± 13.90	104.89-114.75	0.043	0.85
	**female**	51	109.19 ± 15.01	108.39-114.42		
** **2019	**male**	50	109.10 ± 17.08	104.24-113.96	−0.12	0.54
	**female**	55	110.95 ± 13.19	107.37-114.51		
** **2018	**male**	52	103.87 ± 14.70	99.77-107.96	−0.54	0.004
	**female**	79	111.41 ± 13.48	108.39-114.42		
**Medical Major Choice**						
** **Own decision^a^	**male**	105	108.51 ± 15.56	105.50-111.52	−0.25	0.057
	**female**	143	112.14 ± 13.62	109.89-114.39		
** **Others^b^	**male**	30	102.87 ± 15.07	97.23-108.50	−0.19	0.43
	**female**	42	105.61 ± 13.30	101.47-109.76		
**Urban level**						
** **Big city^c^	**male**	19	109.16 ± 13.12	102.84 - 115.48	−0.28	0.36
	**female**	25	112.84 ± 13.17	107.40 - 118.27		
** **Small city^d^	**male**	81	107.28 ± 15.86	103.78 - 110.79	−0.24	0.11
	**female**	109	110.78 ± 13.22	108.27 - 113.29		
** **Rural areas^e^	**male**	35	106.17 ± 16.43	100.53 - 111.82	−0.20	0.37
	**female**	51	109.33 ± 15.31	105.03 - 113.64		
**Internship**						
** **Participated	**male**	106	105.99 ± 16.23	102.86-109.12	−0.40	0.002
	**female**	138	111.90 ± 12.79	109.75-114.06		
** **Not participated	**male**	29	111.90 ± 12.03	107.31-116.47	0.35	0.13
	**female**	47	107.00 ± 15.96	102.31-111.68		
**Clinical courses** ^**f**^						
** **Participated	**male**	106	106.61 ± 15.96	103.54-109.69	−0.33	0.011
	**female**	139	111.51 ± 13.25	109.30-113.74		
** **Not participated	**male**	29	109.62 ± 14.07	104.27-114.97	0.11	0.65
	**female**	46	108.06 ± 15.17	103.56-112.57		
**Number of romantic Relationships**						
** **0	**male**	44	104.29 ± 15.76	99.50-109.08	−0.35	0.059
	**female**	90	109.78 ± 15.34	106.56-112.99		
** **1	**male**	38	107.39 ± 15.28	102.37-112.42	−0.29	0.20
	**female**	39	111.44 ± 12.12	107.50-115.36		
** **2	**male**	30	108.37 ± 16.36	102.26-114.47	−0.34	0.19
	**female**	31	112.70 ± 7.63	109.90-115.51		
** **3	**male**	16	108.63 ± 15.43	100.40-116.84	−0.054	0.88
	**female**	16	109.56 ± 18.78	99.56-119.56		
** ** > 3	**male**	7	117.28 ± 10.66	107.43-127.14	0.55	0.29
	**female**	9	111.00 ± 12.07	101.72-120.28		
**Experience of breakups**						
** **No	**male**	62	105.05 ± 14.49	101.37-108.73	−0.35	0.030
	**female**	103	110.25 ± 15.27	107.27-113.24		
** **Yes	**male**	73	109.14 ± 16.30	105.33-112.94	−0.14	0.38
	**female**	82	111.17 ± 11.73	108.59-113.75		

Note: The table presents the empathy scores (mean ± standard deviation) along with 95% confidence interval across gender stratified by other variables.

### 3.4 Empathy scores across medical major choice stratified by other variables

We also conducted an analysis of the scores across medical major choice based on other variables. The empathy scores of undergraduates who have initially intend to pursue a career in medicine were found to be higher compared to those who did not have such intentions, across all other variables, with statistically significant differences in the fifth year (Grade 2018), female students, rural areas, clinical courses, clinical internship experience, number of romantic relationships greater than 3, and breakup experience. Undergraduate students who originally had a medical volunteer inclination maintained relatively balanced empathy scores across almost all variables, with an average score of approximately 110 points. In contrast, undergraduate students without a medical volunteer inclination showed more fluctuating empathy scores across different variables (Table 3).

**Table 3:** Empathy scores across medical major choice stratified by various factors (n = 320).

Factor	Medical major choice	No. of participants	Empathy score		Effect size	P-value
			Mean ± standard deviation	95% confidence interval		
**Grade**						
** **2020	**own decision** ^**a**^	69	111.67 ± 14.14	108.27-115.06	0.96	0.002
	**others** ^**b**^	15	99.20 ± 11.80	92.66-105.74		
** **2019	**own decision**	78	110.79 ± 15.26	107.35-114.24	0.19	0.40
	**others**	27	107.96 ± 14.81	102.11-113.82		
** **2018	**own decision**	101	109.73 ± 14.36	106.90-112.57	0.41	0.054
	**others**	30	103.97 ± 13.87	98.79-109.14		
**Gender**						
** **Male	**own decision**	105	108.51 ± 15.56	105.50-111.52	0.37	0.080
	**others**	30	102.87 ± 15.08	97.24-108.50		
** **Female	**own decision**	143	112.14 ± 13.62	109.89-114.39	0.48	0.007
	**others**	42	105.62 ± 13.30	101.47-109.76		
**Urbanization level**						
** **Big city^c^	**own decision**	35	111.80 ± 13.69	107.10-116.50	0.22	0.59
	**others**	9	109.11 ± 11.10	100.58-117.64		
** **Small city^d^	**own decision**	147	110.10 ± 14.61	107.71-112.48	0.25	0.16
	**others**	43	106.53 ± 13.78	102.29-110.78		
** **Rural areas^e^	**own decision**	66	111.11 ± 15.02	107.41-114.80	0.90	0.001
	**others**	20	97.95 ± 14.13	91.34-104.56		
**Clinical course** ^**f**^						
** **Yes	**own decision**	188	110.49 ± 14.73	108.38-112.61	0.33	0.033
	**others**	57	105.77 ± 13.94	102.07-109.47		
** **No	**own decision**	60	110.95 ± 14.11	107.30-114.60	0.82	0.006
	**others**	15	99.53 ± 13.71	91.94-107.12		
**Internship experience**						
** **Yes	**own decision**	185	110.38 ± 14.70	108.25-112.52	0.30	0.048
	**others**	59	106.05 ± 14.12	102.37-109.73		
** **No	**own decision**	63	111.25 ± 14.21	107.68-114.83	1.1	0.001
	**others**	13	97.31 ± 11.54	90.34-104.28		
**Number of romantic relationships**						
** **0	**own decision**	98	109.18 ± 16.15	105.95-112.42	0.30	0.14
	**others**	36	104.69 ± 13.85	100.01-109.38		
** **1	**own decision**	65	110.57 ± 13.93	107.12-114.02	0.56	0.096
	**others**	12	103.33 ± 12.02	95.70-110.97		
** **2	**own decision**	47	111.36 ± 13.01	107.54-115.18	0.27	0.38
	**others**	14	107.93 ± 12.05	100.97-114.88		
** **3	**own decision**	26	112.08 ± 14.84	106.08-118.07	0.89	0.035
	**others**	6	96.17 ± 20.62	74.52-117.81		
** > **3	**own decision**	12	116.25 ± 7.69	111.36-121.14	0.70	0.37
	**others**	4	106.25 ± 18.73	76.44-136.06		
**Experience of breakups**						
** **No	**own decision**	124	109.61 ± 15.42	106.87-112.35	0.36	0.052
	**others**	41	104.32 ± 13.72	99.99-108.65		
** **Yes	**own decision**	124	111.60 ± 13.62	109.18-114.02	0.49	0.014
	**others**	31	104.68 ± 14.66	99.30-110.06		

Note: The table presents the empathy scores (mean ± standard deviation) along with 95% confidence interval across gender stratified by other variables.

### 3.5 Association of different factors with JSE-S component scores

The JES-S comprises three components: compassionate care, perspective-taking, and walking in the patient’s shoes. The scores for each component were further analyzed while controlling for various variables. Significant differences in scores were observed among the three components of medical major choice. Scores for medical students with a voluntary selection and inclination towards a medical major were higher in all three aspects compared to the control group, as shown in **Table 4**.

**Table 4:** The association of various variables with three component scores of the JSE-S (n = 320).

Independent factor	Perspective taking	Compassionate care	Walking in the patient’s shoes
**Gender**			
** **Male	57.13	41.16	8.96
** **Female	57.42	43.76	9.48
**Grade**			
** **2020	57.29	43.13	9.01
** **2019	57.40	43.35	9.31
** **2018	57.22	41.81	9.38
**Medical Major Choice**			
** **Own decision^a^	58.06	43.15	9.39
** **Others^b^	54.67	40.99	8.82
**Urban level**			
** **Big city^c^	58.00	43.31	9.93
** **Small city^d^	57.37	42.72	9.18
** **Rural areas^e^	56.77	42.18	9.08
**Clinical course** ^f^			
** **Participated	57.42	42.67	9.29
** **Not participated	56.89	42.62	9.15
**Internship**			
** **Participated	57.55	42.47	9.32
** **Not participated	56.51	43.28	9.08
**Number of Romantic Relationships**			
** **0	56.98	42.14	8.85
** **1	57.59	42.58	9.26
** **2	57.34	43.45	9.77
** **3	56.90	42.59	9.59
** ** > 3	59.12	44.5	10.12
**Experience of Breakups**			
** **No	57.00	42.33	8.95
** **Yes	57.61	43.01	9.58

Note: The table presents average scores for each structure of JSE-three structures based on various variables.

### 3.6 Multivariate logistic regression model

Considering the potential collinearity between clinical courses and internships, we excluded the variable of clinical courses, which is relatively less correlated. Subsequently, using the average score of the JSE-S scale from 320 students as the threshold, empathy scores were transformed into a binary variable, and a multivariate binary logistic regression model was constructed. This predictive model employed the enter method for variable selection, forcibly including seven variables except for clinical courses. The model revealed that medical major choice (p = 0.000) and gender (p = 0.024) exhibited statistically significant differences. Among these, medical major choice (B value, 1.13; OR, 3.11; 95% CI, 1.73–5.58) had the greatest impact on the model, surpassing the influence of gender (B value, 0.54; OR, 1.71; 95% CI, 1.07–2.74). The area under the ROC curve for this model was 0.65 (S1 Table).

## 4 Disscussion

In the process of medical education, factors associated with empathy should not be disregarded. Instead, there should be an in-depth exploration of elements affecting medical student empathy, such as interest in medicine. Nurturing factors conducive to medical empathy and curbing adverse elements as early as possible may have a significantly positive impact on the development of empathy among medical students. In this study, we primarily aimed to identify relevant factors influencing the empathy levels of medical students. The results revealed that two variables—medical major choice and gender—were associated with the empathy levels of medical students. Additionally, the logistic regression model indicated that the impact of medical major choice on empathy was greater than that of the inherent variable, gender. However, the model’s discriminative ability was only moderate (AUC = 0.65), suggesting that the model might not include other important factors influencing empathy. Therefore, it is necessary to explore further additional factors affecting empathy to establish a more accurate predictive model.

Differences in personal qualities between men and women have long been a hotly discussed topic. Numerous studies have consistently demonstrated that women exhibit a greater capacity for empathy than men, both within the general population [28] and health care professionals [16]. Our study aligns with previous research indicating that women tend to display higher levels of empathy than men. The reasons behind this disparity have sparked extensive discussions. Eagly et al categorized the origin of sex differences in human behavior into two primary theories: evolutionary origin theory and social structure origin theory [29]. It’s not surprising that women surpass men in empathetic tendencies due to inherent disparities in social division of labor, maternal investment, and biology [30–32]. All these abovementioned factors may contribute to sex-based variations in neural circuits associated with empathy through neural plasticity described by Doidge [33].

This study found that students who initially had an intention to study medicine exhibited higher empathy scores compared to those without such an intention, regardless of their grade, gender, urbanization level of their home address, internship experience, or romantic relationships (Tables 1 and 3). Additionally, these voluntarily chosen medical students outperformed their counterparts across all three dimensions of the JSE-S (Table 4). Finally, our predictive model also showed that medical major choice was the variable with the greatest impact on outcomes among all variables, surpassing the innate variable—gender (S1 Table). This suggests that an initial interest in medicine may serve as a relatively stable indicator associated with the empathy levels of medical students. Chen and Hojat et al. found that medical students who chose people-oriented specialties had higher empathy scores than those in technology-oriented specialties [34,35]. Their research focused on the choice of secondary specialties after undergraduate medical education, whereas our study concentrates on the interest in medicine prior to undergraduate medical education, emphasizing the intrinsic motivation for pursuing medicine. Interest is a driving factor for learning motivation. In the process of medical education, medical schools should also place emphasis on cultivating students’ interest in medicine.

This study possesses advantages that currently surpass other articles in this field. Firstly, we innovatively explored the correlations between original medical major choice, frequency of romantic relationships, and breakup experiences with empathy scores. Secondly, our research methodology is worthy of reference. After initially using t-tests and ANOVA, we conducted post-hoc t-tests on variables with significant differences to explore their distribution across other variables. Following this, we also employed binary logistic regression to analyze the quantitative relationships between eight factors and empathy scores. Finally, to our knowledge, this study is the first to incorporate initial medical major choice into empathy research. Our findings suggest that medical major choice could serve as a potential indicator of empathy levels among undergraduate medical students, possibly surpassing the inherent variable of gender in its impact. Nevertheless, there are some limitations in this study. Firstly, it is a cross-sectional study, and the influence of cohort effects cannot be completely ruled out. Therefore, longitudinal studies are needed to verify these findings. Secondly, the method used in this study to assess empathy is a self-report scale, and there is no simultaneous objective evaluation based on patients, doctors, or teachers. As a result, there may be discrepancies between self-reported results and actual content.

## 5 Conclusion

This study indicates that medical major choice and gender are predictive factors of empathy ability among medical students. More large-sample studies or qualitative research should be conducted to explore the influencing factors of empathy ability in medical students, with the aim of further enhancing their empathy levels. Additionally, we found that students who initially did not choose a medical major and male students are groups with relatively lower empathy levels. Medical schools should focus on empathy education for these groups in future training programs, aiming to increase their interest in medicine and improve their empathy level.

## Supporting information

S1 File Reliability and validity analysis. This document presents the software-exported results of the reliability and validity analysis of the scale.
(DOCX)

S1 Table Binary logistic regression analysis (Enter). The table illustrates a binary logistic regression analysis of factors influencing empathy levels. Using the average score of the JSE-S scale among 320 students as a threshold, empathy scores were transformed into a binary categorical variable. Those below the average were categorized as having low empathy levels, while those above the average were categorized as having high empathy levels. In order to address the issue of collinearity between clinical practice and clinical courses, we have removed the relatively insignificant clinical courses from our analysis. This predictive model employed the enter method to screen variables, forcibly incorporating seven variables, excluding clinical courses. Additionally, due to the urbanization level being a multicategorical variable, rural areas were taken as the reference level, and the urbanization levels were dummy-coded. Urbanization level (1) refers to the family address in a small city, and urbanization level (2) refers to the family address in a large city. The results indicate that the choice of medical major and gender are the two most significant variables. Among them, the choice of medical major has the greatest impact on empathy, surpassing gender.
(DOCX)
