# Supplementary material for: Empathy in undergraduate medical students: A multivariate cross-sectional study in China
Source: PLoS One. 2025 Nov 5;20(11):e0336143. doi: 10.1371/journal.pone.0336143 (PMC12588497; doi:10.1371/journal.pone.0336143)

**Reliability and validity analysis**

| The corresponding letter abbreviations | Jefferson Scale of Empathy Medical Student version (S - version) |
| --- | --- |
| A | 1. Physicians' understanding of their patients' feelings and the feelings of their patients' families does not influence medical or surgical treatment |
| B | 2. Patients feel better when their physicians understand their feelings |
| C | 3. It is difficult for a physician to view things from patients' perspectives |
| D | 4. Understanding body language is as important as verbal communication in physician-patient relationships |
| E | 5. A physician's sense of humor contributes to a better clinical outcome |
| F | 6. Because people are different, it is difficult to see things from patients' perspectives |
| G | 7. Attention to patients' emotions is not important in history taking |
| H | 8. Attentiveness to patients' personal experiences does not influence treatment outcomes |
| I | 9. Physicians should try to stand in their patients' shoes when providing care to them |
| J | 10. Patients value a physician's understanding of their feelings which is therapeutic in its own right |
| K | 11. Patients' illnesses can be cured only by medical or surgical treatment; therefore, physicians' emotional ties with their patients do not have a significant influence in medical or surgical treatment |
| L | 12. Asking patients about what is happening in their personal lives is not helpful in understanding their physical complaints |
| M | 13. Physicians should try to understand what is going on in their patients' minds by paying attention to their non-verbal cues and body language |
| N | 14. I believe that emotion has no place in the treatment of medical illness |
| O | 15. Empathy is a therapeutic skill without which the physician's success is limited |
| P | 16. Physicians' understanding of the emotional status of their patients, as well as that of their families is one important component of the physician-patient relationship |
| Q | 17. Physicians should try to think like their patients in order to render better care |
| R | 18. Physicians should not allow themselves to be influenced by strong personal bonds between their patients and their family members |
| S | 19. I do not enjoy reading non-medical literature or the arts |
| T | 20. I believe that empathy is an important therapeutic factor in medical treatment |

**Note:** The copyright for the use and distribution of this scale is owned by Jefferson University.

Confirmatory Factor Analysis


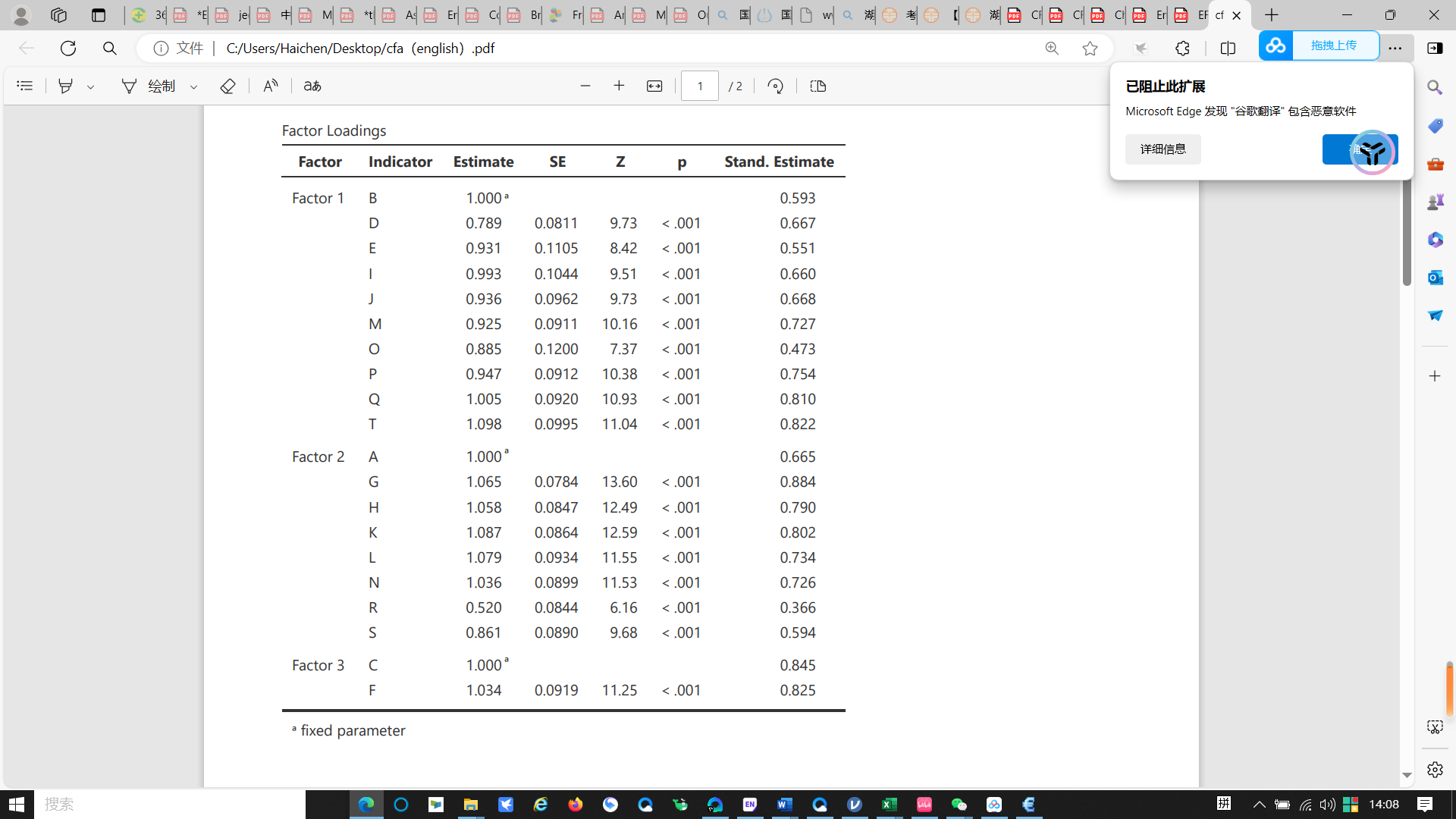


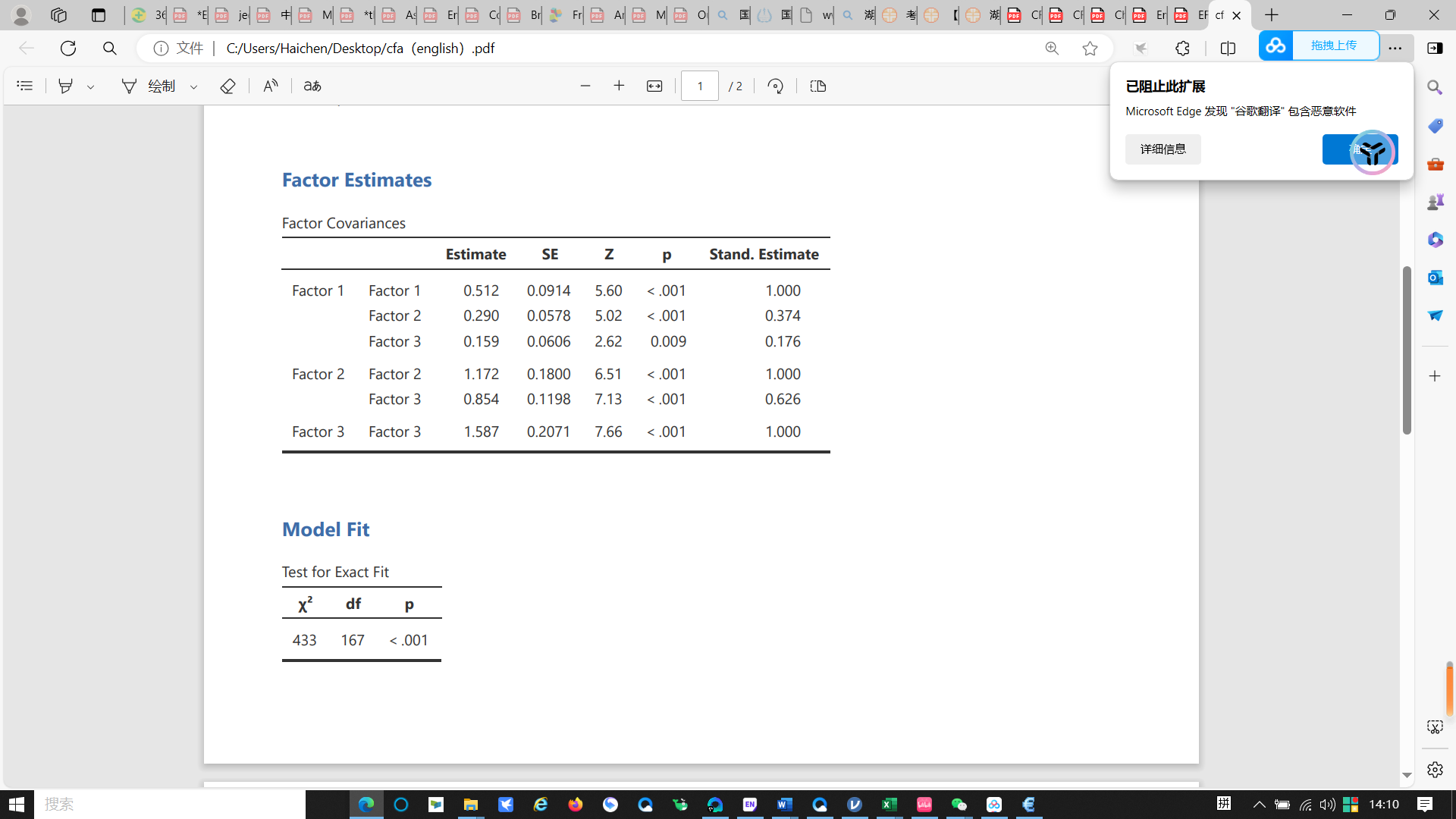


| Fit Measures | | | | | | | | | | | | | |
| --- | --- | --- | --- | --- | --- | --- | --- | --- | --- | --- | --- | --- | --- |
|  | | | | | | **RMSEA 90% CI** | | | |  | | | |
| **CFI** | | **TLI** | | **RMSEA** | | **Lower** | | **Upper** | | **AIC** | | **BIC** | |
| 0.915 |  | 0.904 |  | 0.0706 |  | 0.0624 |  | 0.0788 |  | 18171 |  | 18408 |  |
|  | | | | | | | | | | | | | |

| AVE and CR index results of the model | | |
| --- | --- | --- |
| Factor | Average variance extracted (AVE) | Composite reliability(CR) |
| Factor1 | 0.4635 | 0.8939 |
| Factor2 | 0.5055 | 0.8866 |
| Factor3 | 0.6973 | 0.8217 |


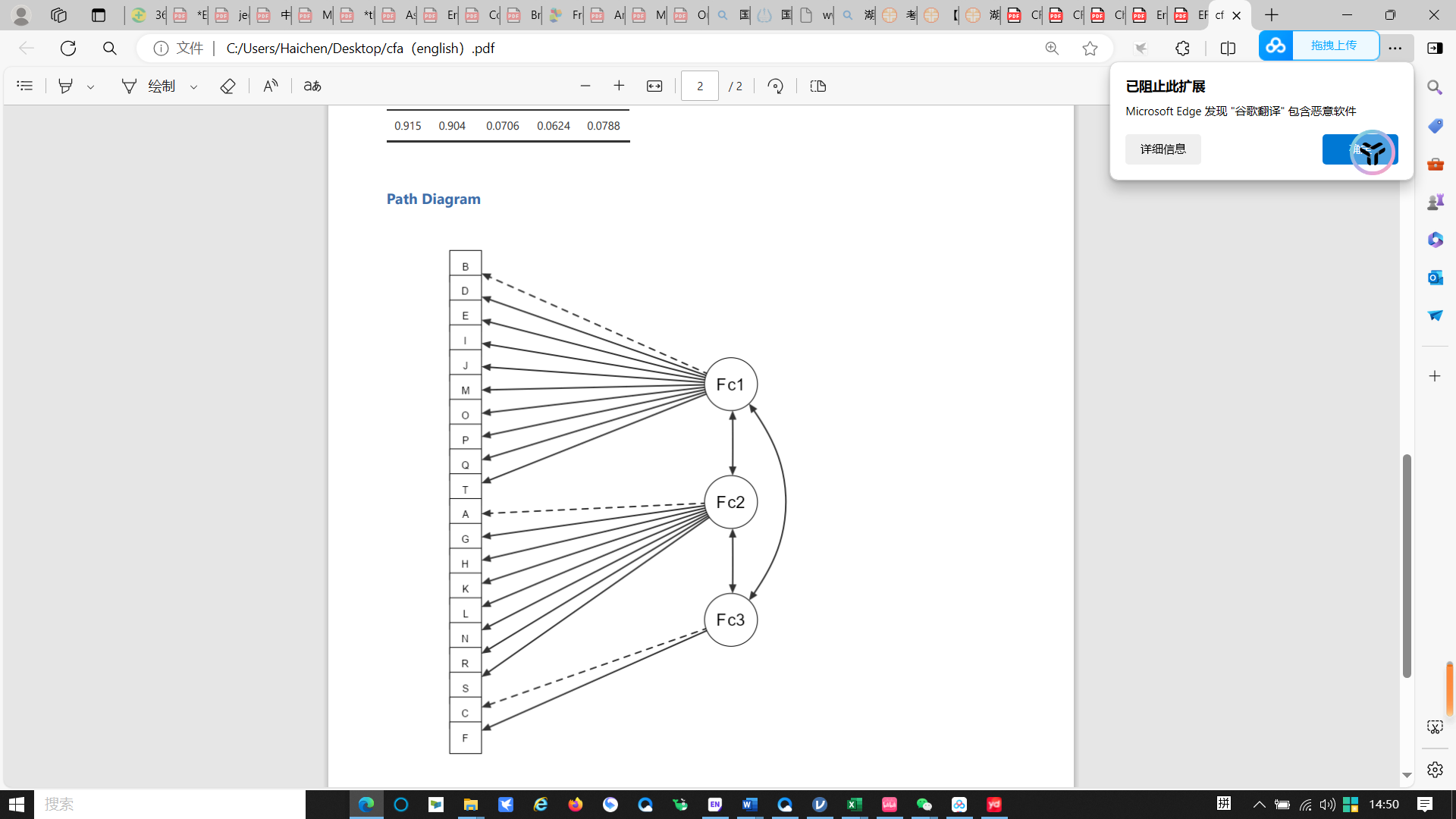


**Correlation Matrix**

| Correlation Matrix | | | | | | | | | |
| --- | --- | --- | --- | --- | --- | --- | --- | --- | --- |
|  | |  | | **factor1** | | **factor2** | | **factor3** | |
| factor1 |  | Pearson's r |  | SQRT (0.4635)  =0.681 |  |  |  |  |  |
|  |  | df |  | — |  |  |  |  |  |
|  |  | p-value |  | — |  |  |  |  |  |
| factor2 |  | Pearson's r |  | 0.317 |  | SQRT (0.5055)  =0.711 |  |  |  |
|  |  | df |  | 318 |  | — |  |  |  |
|  |  | p-value |  | < .001 |  | — |  |  |  |
| factor3 |  | Pearson's r |  | 0.131 |  | 0.557 |  | SQRT (0.6973)  =0.835 |  |
|  |  | df |  | 318 |  | 318 |  | — |  |
|  |  | p-value |  | 0.019 |  | < .001 |  | — |  |

**Plot**


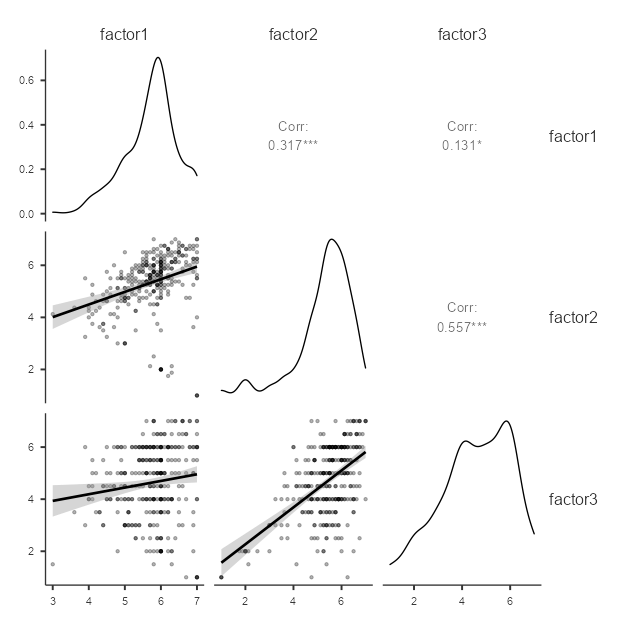

Supplement: S1 File — This document presents the software-exported results of the reliability and validity analysis of the scale. (DOCX) [file pone.0336143.s001.docx]
