# Supplementary material for: Empathy in undergraduate medical students: A multivariate cross-sectional study in China
Source: PLoS One. 2025 Nov 5;20(11):e0336143. doi: 10.1371/journal.pone.0336143 (PMC12588497; doi:10.1371/journal.pone.0336143)
Supplement: S1 Table — The table illustrates a binary logistic regression analysis of factors influencing empathy levels. Using the average score of the JSE-S scale among 320 students as a threshold, empathy scores were transformed into a binary categorical variable. Those below the average were categorized as having low empathy levels, while those above the average were categorized as having high empathy levels. In order to address the issue of collinearity between clinical practice and clinical courses, we have removed the relatively insignificant clinical courses from our analysis. This predictive model employed the enter method to screen variables, forcibly incorporating seven variables, excluding clinical courses. Additionally, due to the urbanization level being a multicategorical variable, rural areas were taken as the reference level, and the urbanization levels were dummy-coded. Urbanization level (1) refers to the family address in a small city, and urbanization level (2) refers to the family address in a large city. The results indicate that the choice of medical major and gender are the two most significant variables. Among them, the choice of medical major has the greatest impact on empathy, surpassing gender. (DOCX) [file pone.0336143.s002.docx]

**S1 table. Binary logistic regression analysis (Enter). (n=320)**

| **Variable** | **B value** | **SE** | **Waldχ^2^** | **P value** | **OR** | **OR(95%CI)** | **AUC** |
| --- | --- | --- | --- | --- | --- | --- | --- |
| **Grade** | 0.25 | 0.22 | 1.32 | 0.25 | 1.28 | 0.84-1.96 | 0.65 |
| **Medical major choice** | 1.13 | 0.30 | 14.48 | 0.000 | 3.11 | 1.73-5.58 |  |
| **Gender** | 0.54 | 0.24 | 5.06 | 0.024 | 1.71 | 1.07-2.74 |  |
| **Urban level** |  |  | 1.38 | 0.50 |  |  |  |
| **Urban level (1)** | -0.37 | 0.35 | 1.10 | 0.30 | 0.69 | 0.35-1.38 |  |
| **Urban level (2)** | -0.45 | 0.40 | 1.28 | 0.26 | 0.64 | 0.30-1.39 |  |
| **Internship** | -0.53 | 0.41 | 1.65 | 0.20 | 0.59 | 0.27-1.32 |  |
| **Number of romantic relationships** | 0.13 | 0.15 | 0.72 | 0.40 | 1.14 | 0.84-1.54 |  |
| **Experience of breakups** | -0.009 | 0.18 | 0.002 | 0.96 | 0.99 | 0.69-1.42 |  |
| **Constant** | -2.70 | 0.83 | 10.55 | 0.001 | 0.07 |  |  |

Note: The table illustrates a binary logistic regression analysis of factors influencing empathy levels. Using the average score of the JSE-S scale among 320 students as a threshold, empathy scores were transformed into a binary categorical variable. Those below the average were categorized as having low empathy levels, while those above the average were categorized as having high empathy levels. In order to address the issue of collinearity between clinical practice and clinical courses, we have removed the relatively insignificant clinical courses from our analysis. This predictive model employed the enter method to screen variables, forcibly incorporating seven variables, excluding clinical courses. Additionally, due to the urbanization level being a multicategorical variable, rural areas were taken as the reference level, and the urbanization levels were dummy-coded. Urbanization level (1) refers to the family address in a small city, and urbanization level (2) refers to the family address in a large city. The results indicate that the choice of medical major and gender are the two most significant variables. Among them, the choice of medical major has the greatest impact on empathy, surpassing gender.
